# Editorial: Macrophages in Liver Disease

**DOI:** 10.3389/fimmu.2020.01754

**Published:** 2020-08-04

**Authors:** Ruchi Bansal, Pranoti Mandrekar, Sujit K. Mohanty, Ralf Weiskirchen

**Affiliations:** ^1^Translational Liver Research, Department of Medical Cell BioPhysics, Technical Medical Centre, Faculty of Science and Technology, University of Twente, Enschede, Netherlands; ^2^Department of Medicine, University of Massachusetts Medical School, Worcester, MA, United States; ^3^Department of Pediatric and Thoracic Surgery, Cincinnati Children's Hospital Medical Centre, Cincinnati, OH, United States; ^4^Institute of Molecular Pathobiochemistry, Experimental Gene Therapy and Clinical Chemistry (IFMPEGKC), RWTH University Hospital Aachen, Aachen, Germany

**Keywords:** liver diseases, macrophage phenotypes, therapeutic targets, treatment strategies, innate immunity

Macrophages constitute a key component of our immune system and play an important role in immune surveillance. Hepatic macrophages are a heterogeneous population of immune cells that mainly comprises of embryonically-derived resident Kupffer cells (KCs), and circulating monocyte-derived macrophages (MoMFs). They play a critical role in disease initiation and progression as well as contribute to disease resolution. Traditionally, macrophages were defined by two broad subsets: classically-activated pro-inflammatory M1 or alternatively-activated anti-inflammatory M2 macrophages. However, it has been recognized that macrophages can differentiate into multiple phenotypes with distinct functions based on the tissue microenvironment.

A review by van der Heide et al. summarizes the current understanding of the hepatic macrophages, their diverse origins and phenotypes, and their role in maintaining homeostasis, progression, as well as in the resolution of liver diseases. Furthermore, the review provides a comprehensive overview of the therapeutic targeting strategies against hepatic macrophages developed for the treatment of liver diseases. Blériot and Ginhoux further elaborated on understanding the heterogeneity of hepatic macrophages. The review briefly discuss the intrinsic and extrinsic factors including metabolic zonation and how they impact cellular phenotypes, functions, and liver physiology. It further provides insights into the recent advances of single-cell transcriptomic approaches, and how they contribute to decipher the liver macrophage heterogeneity and biology.

Liver injury triggers the recruitment of extrahepatic monocytes that replenish the pool of hepatic macrophages upon resident KCs depletion or damage. This heterogeneous population of macrophages are involved in the pathogenesis of alcoholic liver disease (ALD), non-alcoholic fatty liver disease (NAFLD), hepatitis B virus/hepatitis C virus (HBV/HCV), and hepatocellular carcinoma (HCC). Dou et al. summarize current knowledge about the role of tissue-resident and recruited macrophages in the pathogenesis of different etiological liver diseases. The review further describes the existence of multiple macrophage origins and phenotypes, their identification markers and roles in disease pathogenesis, and how this knowledge can be translated into future therapies. Oates et al. highlight the landscape of mechanisms underlying macrophage dynamics, macrophage interplay with other cells/tissues, and immunometabolism that collectively contribute to NAFLD progression. Since macrophage-driven inflammation is intricately linked to various metabolic pathways, the potential benefits to be gained from understanding the interplay between metabolic and inflammatory pathways in macrophages are immense.

In an original research article, Schierwagen et al. investigated the involvement of macrophage-inducible C-type lectin (mincle), expressed on macrophages, in different stages of chronic liver disease (CLD). The authors showed increased mincle expression that correlated with disease severity as examined in rodent models of cirrhosis, non-alcoholic steatohepatitis (NASH) and acute-on-chronic liver failure (ACLF), and in patients with NASH and cirrhosis, and that undergoing bariatric surgery. They further showed that mincle activation using mincle agonist, trehalose-6,6-dibehenate, significantly increased collagen production in ApoE-deficient high-fat Western diet-induced NASH mouse model and further confirmed in cirrhosis and ACLF animal models. These findings suggested that mincle expressed on macrophages contribute to inflammation and fibrosis, when the intestinal barrier becomes leaky, during advanced stages of CLD. Macrophages (and innate immunity) also play an important role in the pathogenesis of biliary atresia (BA), a devastating cholangiopathy of infancy progressing to end-stage liver disease often requiring liver transplantation. A review by Ortiz-Perez et al. provides a comprehensive overview of BA immunopathogenesis and the intricate mechanisms involved in the disease pathogenesis. The authors further highlighted the challenges such as lack of suitable experimental models that hinder the deeper understanding of the disease, etiology, and development of new therapies.

Hepatic macrophages interact with multiple cell types in the liver including hepatocytes, cholangiocytes, hepatic stellate cells (HSCs), liver sinusoidal endothelial cells (LSECs), platelets, and other immune cells. A review by Shan and Ju describes the impact of tissue microenvironmental factors that determine the phenotype and function of hepatic macrophages, and the crosstalk between hepatic macrophages and other hepatic cells, in regulating the extents of liver injury, repair and disease progression. For instance, macrophages secrete factors, can physically interact with vasculature to assist the formation of complex vascular networks, and activate HSCs and LSECs, and promote fibrogenesis and angiogenesis, respectively. Conversely, HSCs and LSECs (and other hepatic cells) secrete chemotactic factors increasing intra-hepatic macrophage infiltration. Ramirez-Pedraza and Fernández reviewed the molecular and cellular crosstalk between macrophages and angiogenesis, and provides detailed insights into the contribution of macrophages to liver steatosis, fibrosis, cirrhosis, HCC, and extrahepatic complications. Targeting angiogenesis-inflammation axis therefore can be an interesting approach for the treatment of liver diseases.

Macrophages also play a central role in keeping the balance of immunity and tolerance. During early HCC development, fibro-inflammation driven by pro-inflammatory macrophages (and HSCs) provide a microenvironment permissive for tumor initiation, while at advanced HCC, macrophage switching to immunosuppressive tumor-associated macrophages (TAMs) support tumor progression and malignancy. The crosstalk between tumor cells (the “seeds”) and microenvironment (the “soil”) play an important role in tumor development and metastasis. A review by Sällberg and Pasetto focuses on macrophages and other key cellular components of the liver and tumor microenvironment, their role in controlling the balance between tolerance and activation, and the potential therapeutic interventions to tilt the balance against liver cancer progression. Hepatic macrophages, particularly KCs, are essential for maintaining homeostasis by scavenging bacteria and cellular debris, and thereby induce immunological tolerance. An inefficient efferocytosis mechanism impairing the clearance of cellular debris results in subsequent loss of tissue homeostasis. Horst et al. describe how efferocytosis in hepatic macrophages regulate tissue homeostasis and regeneration in liver diseases. It is essential to understand the tempo-spatial contribution of macrophages and efferocytosis mechanisms in different etiological liver diseases, for the development of potential interventions against liver diseases.

Besides efferocytosis, macrophages facilitate resolution of liver fibrosis [characterized by excessive accumulation of extracellular matrix (ECM) proteins] by producing matrix-degrading enzymes (matrix metalloproteinases, MMPs) that degrade fibrotic ECM. Upon liver damage, macrophages induce HSCs trans-differentiation into proliferative, fibrogenic myofibroblasts that secrete large amounts of ECM proteins, predominantly fibrillar collagens, and ECM crosslinking enzymes, lysyl oxidase-like 2 (LOXL2), that stabilize ECM components. Klepfish et al. demonstrated that a novel anti-LOXL2 monoclonal antibody (GS341) ameliorated liver fibrosis *in vivo*. Mechanistically, anti-LOXL2 antibody inhibited LOXL2-mediated collagen crosslinking and facilitated the recruitment of so-called scar-associated MoMFs (SAMs) expressing a unique repertoire of collagenolytic MMPs (in particular MMP-14) to the proximity of collagenous fibrotic fibers. These findings suggest that therapies augmenting the recruitment of collagenolytic macrophages and/or polarization of macrophages into collagenolytic macrophages might be an interesting approach to reverse fibrosis and facilitate endogenous liver regeneration.

MoMFs recruitment is regulated by monocyte chemotactic protein 1 (MCP-1 or CCL2). Queck et al. investigated intrahepatic expression and circulating levels of MCP-1, and its correlation with monocyte infiltration and severity of liver diseases. Using rodent models of liver cirrhosis and ACLF, authors showed an elevated hepatic expression of CCL2 along with increased F4/80-positive macrophages in the liver. In human liver explants and ACLF patients, hepatic transcription levels of CCL2 correlated with the MELD score, and higher portal and hepatic vein levels of MCP-1 correlated with Child-Pugh score, respectively. This study concluded that MCP-1 circulating levels, derived from the injured liver, reflect the intra-hepatic macrophages and correlate with severity of liver disease.

An interesting review by Shwartz et al. describes an emergence of the teleost zebrafish, an attractive new vertebrate model to study liver macrophages. Authors summarize the origin and functions of macrophages in the livers of zebrafish models of ALD, NAFLD, HCC, and liver regeneration. The review discusses how macrophages in zebrafish models can be compared with that described in mammals and highlights the advantages and challenges of using zebrafish models to study liver macrophages.

Colino et al. summarizes the different types of passive (driven by anatomical and physiological features) and active (using specific ligands) targeted nanoparticle (NP) systems for macrophage recognition and drug targeting. To design NPs, NPs biocompatibility, degradability, toxicity, *in vivo* pharmacokinetics and drug release should be contemplated. Furthermore, recognition mechanisms by macrophages must be investigated considering the changes in the microenvironment that can influence the macrophage phenotype and impact NPs uptake. The authors further present the physiological-based pharmacokinetic (PBPK) model to characterize the biodistribution of NPs.

Altogether, this special issue presents a series of 9 reviews, 2 mini-reviews, and 3 original articles focusing on the understanding of macrophages and/or innate immune system in liver diseases. It also highlights the intricacies of distinct macrophage phenotypes at different stages of the diverse etiological liver diseases and provides a comprehensive overview of the therapeutic targets and macrophages targeting approaches for the treatment of liver diseases.

## Author Contributions

RB drafted and all the other authors read and revised the editorial. All the authors contributed equally to the review and editorial process for this collection.

## Conflict of Interest

The authors declare that the research was conducted in the absence of any commercial or financial relationships that could be construed as a potential conflict of interest.

